# A framework for culturally adapting mental mHealth apps

**DOI:** 10.3389/fdgth.2026.1698145

**Published:** 2026-05-04

**Authors:** Noorah Ibrahim S. Alnaghaimshi, Michael Proeve, Scott R. Clark, Mathias Baumert

**Affiliations:** 1 Discipline of Biomedical Engineering, School of Electrical and Mechanical Engineering, The University of Adelaide, Adelaide, SA, Australia; 2 Department of Computer and Information Science, Majmaah University, Al-Majmaah, Riyadh Region, Saudi Arabia; 3 School of Psychology, Faculty of Health and Medical Sciences, The University of Adelaide, Adelaide, SA, Australia; 4 Discipline of Psychiatry, School of Medicine, The University of Adelaide, Adelaide, SA, Australia; 5 Department of Technical Physics, University of Eastern Finland, Kuopio, Finland

**Keywords:** adaptation, CBT, co-design, cognitive behaviour therapy, mHealth apps

## Abstract

Mobile health (mHealth) apps are increasingly deployed for evidence-based mental health interventions, broadening access to care. While effective, Internet-based Cognitive Behavioural Therapy, delivered via web or app, frequently overlooks ethnic minority and migrant populations. Effective cultural adaptation of mHealth apps is critical to their impact and accessibility; however, existing frameworks often lack specific guidance for digital contexts, relying on superficial adjustments or omitting the explicit integration of religious factors. Our novel framework fundamentally departs from prior models by embedding cultural responsiveness throughout the entire digital development lifecycle of mHealth apps, rather than treating it as a peripheral concern. This comprehensive approach is structured across four interconnected layers: adapting the therapeutic foundation (explicitly incorporating religious considerations), culturally grounding features and reframing standard tools, tailoring content and messaging, and optimizing UX/UI design. Central to this framework is a participatory co-design and prototyping methodology that ensures deep cultural insights, including religious and spiritual dimensions, are profoundly integrated from the earliest stages of development. This framework thus offers a practical, inclusive, and ethical blueprint for designing culturally relevant digital health products and can be applied and tested in future work through iterative co-design workshops, user-centred prototyping, and the development of culturally tailored digital interventions, with ongoing evaluation of their effectiveness and acceptability in diverse populations.

## 1 Introduction

Mobile health (mHealth) apps are effective at reducing symptoms of mental health conditions such as depression and anxiety (1, 2). By improving public access to high-quality remote mental health care in regions where limited local resources restrict the face-to-face option or where there is a stigma against direct engagement, mHealth apps offer an effective tool for tackling mental health challenges (3). mHealth apps can address several gaps in mental health care, from enhancing patient engagement and facilitating treatment processes to providing immediate support and addressing stigma (4). Apps can provide scalable, immediate, and low-cost care tailored to the individual, promoting the fair distribution of mental health care resources (5). While mHealth apps are becoming increasingly popular, their applicability and impact across ethnic groups may be limited by a one-size-fits-all design (6). There is growing recognition of the importance of culturally adapting digital mental health interventions to ensure their effectiveness and accessibility for racially and ethnically minoritised communities (7).

Since culturally adapting digital interventions can be complex, time-consuming, and costly (8), user-centred intervention development that integrates and emphasises cultural context is recognised as crucial for relevance and effectiveness (9). Co-design, a key method in user-centred intervention development, is beneficial for developing mHealth interventions that are acceptable and feasible for minority and indigenous communities (8). Eliciting the specific needs and challenges of racial and ethnic groups in the co-design of mHealth can enhance app effectiveness, usability, and engagement, ultimately leading to improved adoption and long-term use (10).

While co-design has gained recognition by shifting the focus of intervention development from a top-down process to one that prioritises the experiences, preferences, and needs of end-users, there is still a critical need for a guiding model or framework that incorporates cultural responsiveness and digital delivery. Existing frameworks tend to focus on either cultural adaptation or technological implementation, but not both (9). A new integrated model is required to guide the development of culturally adapted and digitally accessible interventions, ensuring cultural responsiveness from the outset (9). Here, we propose a framework for developing culturally relevant and practical mHealth apps that bridge gaps in the current frameworks.

## 2 CBT as the core of adapted mHealth intervention

Cognitive Behavioural Therapy (CBT), the dominant therapeutic framework in mHealth apps, includes evidence-based strategies for context engagement, attention change, and cognitive change (11), which are implemented within structured therapeutic content to support emotional well-being, redirect focus, and challenge negative thinking patterns (11). CBT is a widely used psychotherapy influenced by European-American cultural values (12) designed to reduce psychological distress and dysfunction by examining how individuals' thoughts, feelings, and behaviours contribute to their problems (13). Online or Internet-based Cognitive Behavioural Therapy (iCBT) is effective at expanding access to mental health services for populations with limited access to face-to-face mental health care (14). Studies show that iCBT has beneficial treatment effects for anxiety and depression among different ethnic groups and minorities (15, 16). However, a recent review found that there is under-reporting of racial and ethnic diversity across studies, with only 27% of the 62 randomised controlled trials (RCTs) of iCBT for depression recording data on race and only 19% included ethnicity (17). Failure to address the unique cultural and contextual needs of these populations beyond language translation or surface-level visual appearance in therapeutic models, communication style, and user interaction patterns can reduce the appropriateness and effectiveness of the intervention and perpetuate or exacerbate health disparities.

Despite these limitations, CBT processes are still relevant across cultures for the treatment of depression. For example, CBT's features of a structured educational approach, emphasis on reinforcing social supports, short-term, directive nature and focus on problem-solving and practical skills, resonate with collectivist cultures (18, 19). CBT aligns with several religious principles of Islam, for example, emphasizing the duty of self-guidance and improvement and concentrates on conscious thought in the here and now (20, 21). There are also significant differences, including Western psychotherapy's emphasis on individuality over the collective through notions of “self-esteem”, “self-actualization”, and “self-statements” (18, 20, 22).

Within the broader field of Cognitive Behavioural Therapy (CBT) adaptation, two distinct yet complementary bodies of research have emerged: **Culturally Adapted CBT (CA-CBT)** and **Religiously Adapted CBT (R-CBT)**. These approaches aim to enhance the relevance, acceptability, and effectiveness of CBT by tailoring it to specific cultural and spiritual contexts. CA-CBT modifies evidence-based treatments to align with the language, cultural norms, and contextual understanding of mental health within a target population (6), which has been shown to significantly boost effectiveness, engagement, trust, and overall treatment outcomes (23), particularly in collectivist cultures (24). R-CBT, on the other hand, integrates clients' religious and spiritual beliefs into the therapeutic process, recognizing these as significant sources of healing and support for religious communities (25, 26). This approach ensures that therapy techniques are relevant, acceptable, and culturally appropriate for the user by incorporating spiritual activities and faith-based rituals, rather than solely self-oriented goals (27–29). For example, in a collectivist setting, behavioural activation may be oriented towards family-supported tasks (CA-CBT) and spiritual activities, such as faith-based rituals (R-CBT). This profound structural adaptation ensures that the therapy technique is relevant, acceptable, and culturally appropriate for the user (30–32).

**Figure 1:**
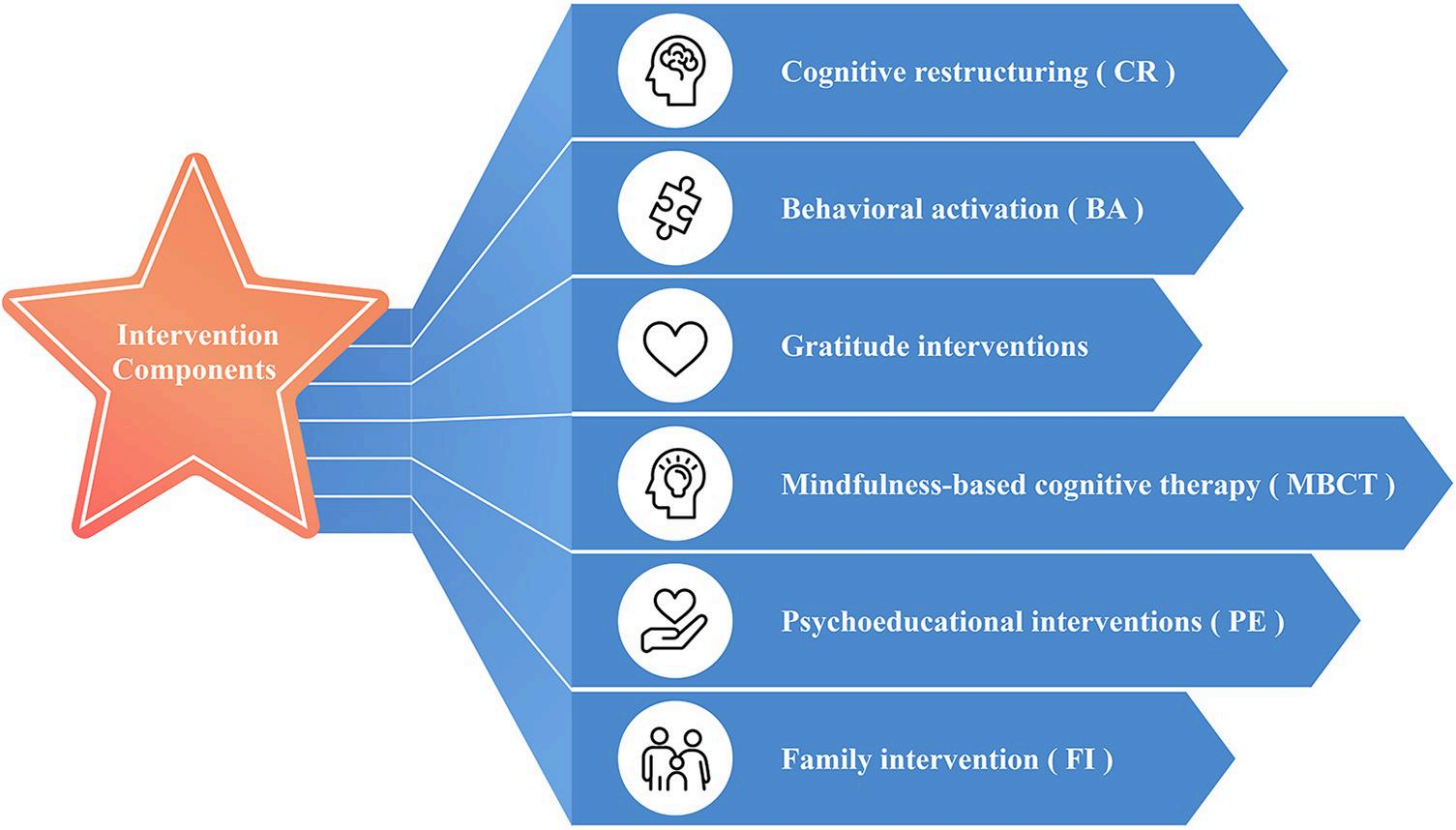
Description of intervention techniques can be delivered via mental m-health.

Given CBT is the therapeutic model adopted for CA-iCBT and R-iCBT, the model's elements and methods need to be discussed. Of these, several CBT-related methods could be implemented, providing the intervention remains evidence-based and meets its primary therapeutic objectives (Figure 1).

Cognitive restructuring (CR) is a key component of Cognitive Behavioural Therapy (CBT) aimed at identifying dysfunctional beliefs and faulty or negative thoughts that arise in response to life events or sources of distress and then guiding patients through cognitive restructuring to challenge and replace these unrealistic thoughts with constructive ones, resulting in a shift in attitude and a more constructive mindset (33). CR is an effective technique to alleviate depressive symptoms among ethnic groups (34, 35). Indeed, CR was recommended to help individuals challenge and modify their harmful interpretations of cultural traditions and teachings, such as the idea that suffering or self-destructive behaviour is necessary to become more devout or spiritually superior (aspects of Buddhism, Confucianism or Taoism) (36). Familiar cultural terminology and metaphors to frame the concept of CR could be used to conform with CA-iCBT and R-iCBT, with an emphasis on balance, and by using, for example, a religious context to emphasise hope, forgiveness, and compassion to help individuals view challenging situations as opportunities for growth. Furthermore, users could adopt CR with family or friends to enhance individual learning and generate a collective awareness of maladaptive thought patterns and their impact.

Behavioural Activation (BA), a component of cognitive-behavioural therapy, is an effective stand-alone psychological treatment for mild to moderate depression (37–40). BA is ideal for ethnic groups, given its contextual and idiographic approach, and as guided self-help, BA has been shown to reduce depressive symptoms (41, 42). Guided activity, a BA strategy, encourages patients to engage in activities that provide a sense of accomplishment and joy (43). Improving engagement, cost-effectiveness, and accessibility would help establish culturally appropriate guided activities that prioritise tasks aligning with social and religious values and effectively involve family members (44, 45). For CA-iCBT, guided activities could integrate communal gatherings, traditional crafts, or shared meals with extended family. R-iCBT examples might include participating in religious festivals, engaging in charitable acts, or daily prayer.

Another technique practised is gratitude, a cognitive-affective state generally associated with the perception that one has received a personal advantage not consciously sought after, deserved, or gained, but rather because of the good intent of another person (46). According to Emmons and Stern (46), gratitude is based on two pieces of information processed by an individual: (a) an affirmation of goodness or “good things” in one's life and (b) recognising that the reasons for this goodness lie outside the self. Gratitude is associated with greater life satisfaction, improved self-esteem, and overall well-being (47, 48). For many religious-based communities, gratitude is strongly linked to subjective well-being and has been revealed to be an essential component of meaning-making and coping strategies (49).

Mindfulness-based cognitive therapy (MBCT) helps decrease depression by promoting mindfulness skills, such as fostering an attitude of acceptance and living in the present moment (50). MBCT helps individuals become aware of their thoughts and feelings and change how they respond to these thoughts (51), fundamental commonalities shared with, for example, Islamic tenets (52). Positive experiences with MBCT were found to lead to new referrals to mental health services (53). Mindfulness interventions can be tailored to the user's religiosity and personal preferences by incorporating religious rituals and content, allowing users to opt in or out of spiritual and religious components.

Psychoeducation (PE) is a fundamental element of psychotherapy. It provides information and resources to understand risk factors, symptoms, relapse signatures, treatments and their side effects, and lifestyle modifications to help manage their illness (54, 55). Integrating an educational component into an intervention can help minimise self-stigma and improve attitudes towards help-seeking (56, 57), which is especially important in collectivist societies. For CA-iCBT, psychoeducation can frame mental health challenges within a community context, emphasising collective well-being and how mental health can affect not only the individual but also their family and social networks. Modules can also use culturally familiar metaphors or concepts to explain mental health conditions, such as traditional narratives for depression, making complex ideas more accessible and less stigmatising by connecting them to existing cultural knowledge. For R-iCBT, psychoeducation can integrate religious teachings that frame seeking help as an act of faith-encouraged self-care and a responsibility towards oneself and one's community.

Family is the primary social institution in human societies. Different models/approaches for family interventions (FI) in mental health disorders have been developed, and they include family therapy, family management, psychoeducation, communication skills, and problem-solving skills (58–61). For example, family psychoeducation (FPE) can improve patients' attitudes towards medications, reduce global disability from depression, and improve relationships with relatives, reducing family burden, notably in the sub-dimensions of ‘worrying’ and ‘urging’ (62). For Culturally Adapted Internet-based Cognitive Behavioral Therapy (CA-iCBT), interventions could include modules on culturally-informed thought restructuring to help families identify and challenge cultural traditions that might contribute to stigma or harmful interpretations of mental health. Alternatively, CA-iCBT could use scenarios designed to address perceived impact of seeking therapy on family reputation, guiding families to solutions that foster understanding, reduce shame, improve cohesion, and encourage open dialogue. In the context of Religiously Adapted Internet-based Cognitive Behavioral Therapy (R-iCBT), this could involve structured exercises that engage families in shared spiritual practices, fostering collective support and a sense of communal healing.

## 3 Cultural adaptation framework for CBT-based mHealth apps

Several frameworks (12, 63–68) have been proposed to guide the cultural adaptation of psychological interventions, ensuring their relevance and effectiveness across diverse populations. Barrera and Castro's framework (68) offers a systematic approach, defining cultural equivalence through tests of engagement, action theory, and conceptual theory to justify adaptations. Engagement refers to the ability of interventions to reach and involve potential participants successfully. Action theory focuses on the treatment's ability to change mediating variables, which are the mechanisms through which the treatment achieves desired outcomes. Conceptual theory, on the other hand, examines the relationships between these mediators and the ultimate outcomes (68). This framework (68) also outlines a phased process for developing adaptations, including information gathering, preliminary design, testing, and refinement. The Ecological Validity Model (64, 66) focusses on adapting interventions across eight dimensions: language, persons, metaphors, content, concepts, goals, methods, and context. Resnicow and colleagues (65) distinguish between surface structure adaptations, which match observable characteristics, and deep structure adaptations, which incorporate underlying cultural influences on health behaviors. Hinton and Patel (67) further illustrate these principles by detailing how CBT can be adapted through strategies such as explanatory model bridging, cultural grounding, and contextual sensitivity, presenting a transdiagnostic model of distress and outlining specific intervention modifications. A newer conceptual framework (63) emphasizes cultural concepts of distress, treatment components, and treatment delivery, advocating for adaptations rooted in local explanatory models and idioms of distress. Complementing these, an evidence-based framework (12) for culturally adapting CBT outlines a systematic process of information gathering, guideline development, and iterative testing to make minor yet impactful adjustments to CBT techniques and delivery, ensuring cultural competence and patient engagement.

**Figure 2:**
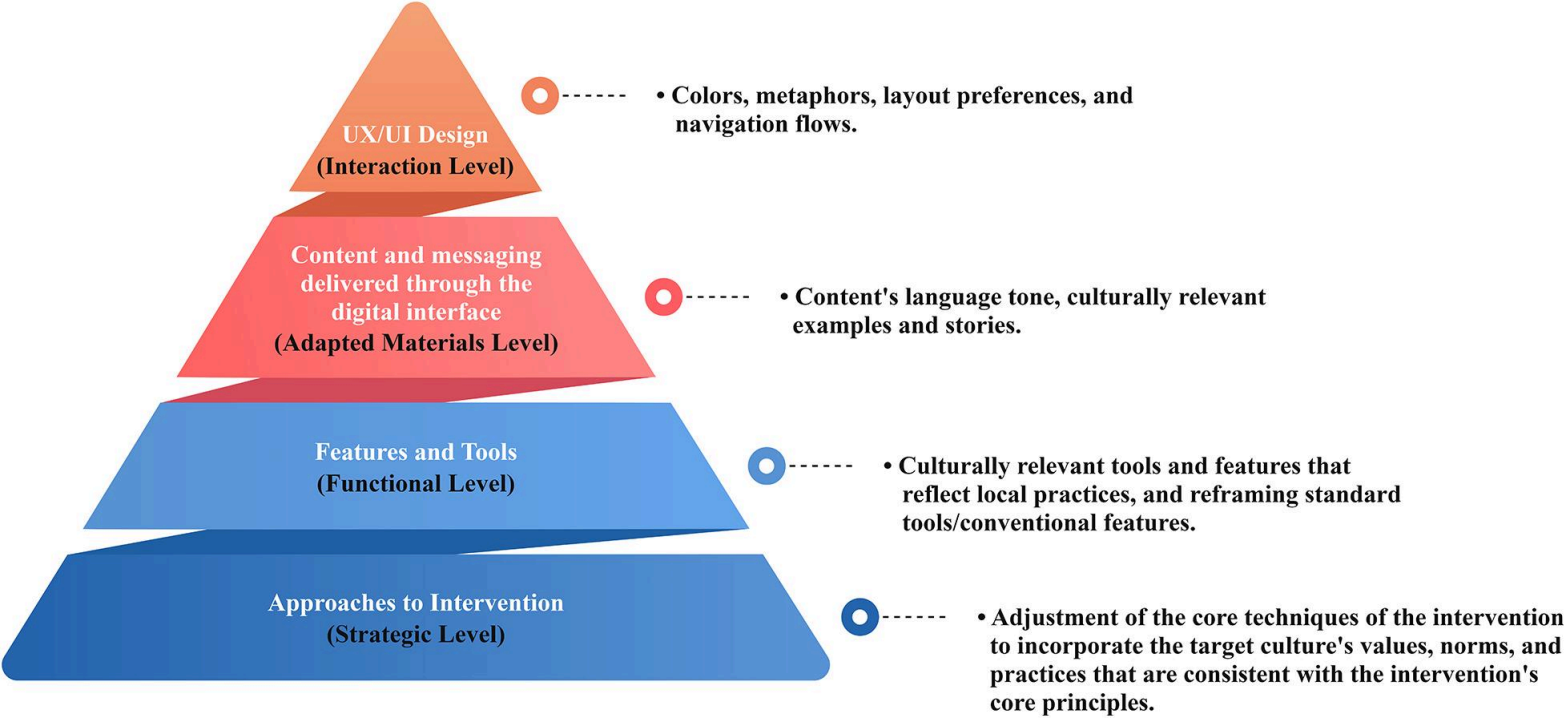
Cultural adaptation layers of the proposed digital CBT intervention.

Existing frameworks (12, 63–68) for cultural adaptation largely focus on in-person or analog delivery, offering limited guidance for integrating cultural and religious elements into digital interface design, navigation, feature logic, and information communication for app development. To address this gap, we propose a new four-layered framework for Culturally Adapted iCBT (CA-iCBT), which includes religious (R-CBT) considerations (Figure 2): 1) cultural adaptation of the therapeutic foundation; 2) culturally grounding features and reframing standard tools; 3) content and messaging adaptation; and 4) UX (User Experience)/UI (User Interface) adaptation. This framework is built on insights from previous studies and established adaptation models (12, 67, 69–75), with a participatory, community-led co-design approach central to all levels.

Our model explicitly addresses the digital context, a gap in prior frameworks (12, 63–68). Central is cultural responsiveness from the therapeutic foundation to digital interaction, offering multi-layered guidance beyond content and messaging to include culturally grounded features, tools, and dedicated UX/UI adaptation. A unique contribution is its integration of Religiously Adapted CBT (R-CBT) within the therapeutic foundation. This acknowledges the profound impact of religious and spiritual beliefs on mental health and coping, enabling nuanced adaptation for faith-based communities, a detail often less explicit in broader cultural models.

Our framework (see Figure 2**)** aims to adapt CBT techniques to align with the target population's beliefs, norms, and coping styles, establishing a clinically sound and culturally relevant foundation. In this way, it is designed to help developers to critically evaluate which strategies or techniques are culturally suitable, and which may require reframing. Once the core techniques are adapted, existing digital tools may need to be refined or new features developed to reflect local practices and culturally grounded strategies. Next, it is important to adapt the content and messaging delivered, including language and terminology, to promote CBT concepts that are understandable and align with users' real-life contexts, which may, in turn, reinforce user engagement. Finally, adapting surface elements, such as culturally relevant visuals, familiar metaphors, navigation flow, and interface layout, aims to make the intervention intuitive and engaging. Positioning UI/UX adaptation as the final stage anchors it as a surface-level integration that reflects and reinforces all prior adjustments. We discuss each of these layers below.

### 3.1 Cultural and religious adaptation of the therapeutic foundation (approaches to intervention)

**Figure 3:**
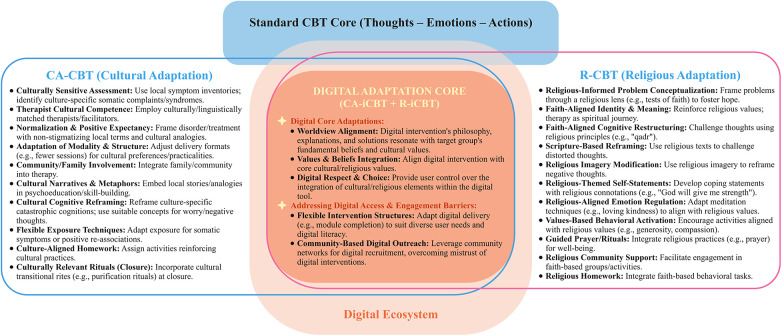
Integrating cultural, religious, and digital adaptations in CBT.

Our first layer of intervention development focuses on adjusting the therapeutic approach to reflect cultural values and beliefs. It entails modifying therapeutic goals, assumptions, and processes to reflect users' cultural ideas about mental health and healing. Previous studies (25, 26, 54–79, 69) proposed different strategies for Culturally Adapted CBT (CA-CBT) and Religiously Adapted CBT (R-CBT) (Figure 3). This involves employing culturally sensitive assessments and therapists, utilising non-stigmatising local terminology, and adapting delivery modalities and structures to overcome practical barriers inherent in diverse communities. Key strategies include involving family and community members, embedding cultural narratives and metaphors for psychoeducation, and applying cultural cognitive reframing to address culture-specific cognitions. Furthermore, CA-CBT meticulously adapts exposure techniques and homework assignments to align with cultural practices and can incorporate culturally relevant rituals for therapeutic closure. In parallel, R-CBT (as shown in Figure 3), integrates clients’ religious and spiritual beliefs by framing life problems through a faith-based lens to cultivate hope and meaning, reinforcing a faith-aligned identity, and employing religious principles and scriptures for cognitive restructuring to challenge maladaptive thoughts. This adaptation further extends to developing religious-themed self-statements for coping, aligning emotion regulation techniques (such as meditation) with spiritual values, and encouraging values-based behavioural activation through religious activities and community engagement. The approach also incorporates guided prayer and rituals, recognising their role in spiritual well-being and discouraging passive coping, while ensuring the therapeutic process remains congruent with the client's spiritual framework.

Building on previous studies (6, 25, 26, 54–85, 69) (as shown in Figure 3), the first is the application of these cultural and religious **Core Adaptations** to the digital ecosystem, involving a multi-faceted approach that ensures alignment with the user's worldview, values, and beliefs, such as addressing individualistic vs. collectivistic perspectives. Second is **Addressing Digital Access & Engagement Barriers**, crucially involving flexible intervention structures and resource optimization to overcome practical obstacles in digital delivery. Also shown in Figure 3, **Digital Core Adaptations** delve into the underlying psychological and spiritual frameworks that imbue the intervention with meaning. *Worldview Alignment* ensures the digital intervention's philosophy and mental health explanations align with the target user's fundamental beliefs about themselves, the world, and the causes/cures of distress. This involves recognizing diverse cultural and religious understandings of mental health issues and healing and integrating cultural and religious coping strategies. *Values & Beliefs Integration* weaves the user's fundamental cultural and religious/spiritual values, as well as their community's priorities, into the narratives and activities presented by the intervention. For *Digital Respect and Choice,* users can select how religious or spiritual elements are integrated into their digital experience, ensuring it aligns with their personal comfort and beliefs. **Addressing Digital Access & Engagement Barriers** means overcoming practical challenges in digital delivery. This can be achieved through *Flexible Intervention Structures*, such as designing interventions with fewer modules or sessions. Furthermore, *Community-Based Digital Outreach* could build trust and reach diverse populations by utilizing trusted community networks for recruitment and promotion.

### 3.2 Culturally grounding features and reframing standard tools (features and tools)

The second layer provides culturally relevant tools and features within digital interventions, informed by cultural understandings of well-being, motivation, communication, and local healing practices. It integrates cultural values into the user experience in ways that extend beyond aesthetics or traditional interaction design. For example, in collectivist culture contexts, providing family-focused educational resources highlights the value of relational support and collaborative healing, thereby reducing stigma (86). Similarly, combining spiritual practice with therapeutic procedures can confirm the user's belief system (69). Likewise, reinterpreting standard digital elements through a cultural perspective, rather than assuming they are neutral or universally applicable, is crucial to maintaining cultural relevance and boosting engagement. For example, reminder systems can shift from compliance-driven nudges to affirming messages grounded in spiritual practices, family-invoking prompts, or communal values. Messaging tailored to demographic, behavioural, and cultural contexts has been shown to have a much higher impact than generic messages (87).

### 3.3 Content and messaging adaptation (content and messaging delivered through the digital interface)

The third layer centres on adapting digital content, which could be critical to shaping the user's therapeutic experience, particularly in the absence of an actual therapist. In this context, “content” includes educational materials, recommendations and guidance, messages, narratives, stories, labels, prompts, and multimedia components. According to a recent systematic review (56), adapting message framing and intervention content to users' linguistic norms, symbolic references, and everyday experiences while maintaining therapeutic fidelity increases engagement through culturally grounded metaphors, symbols and expressions.

**Figure 4:**
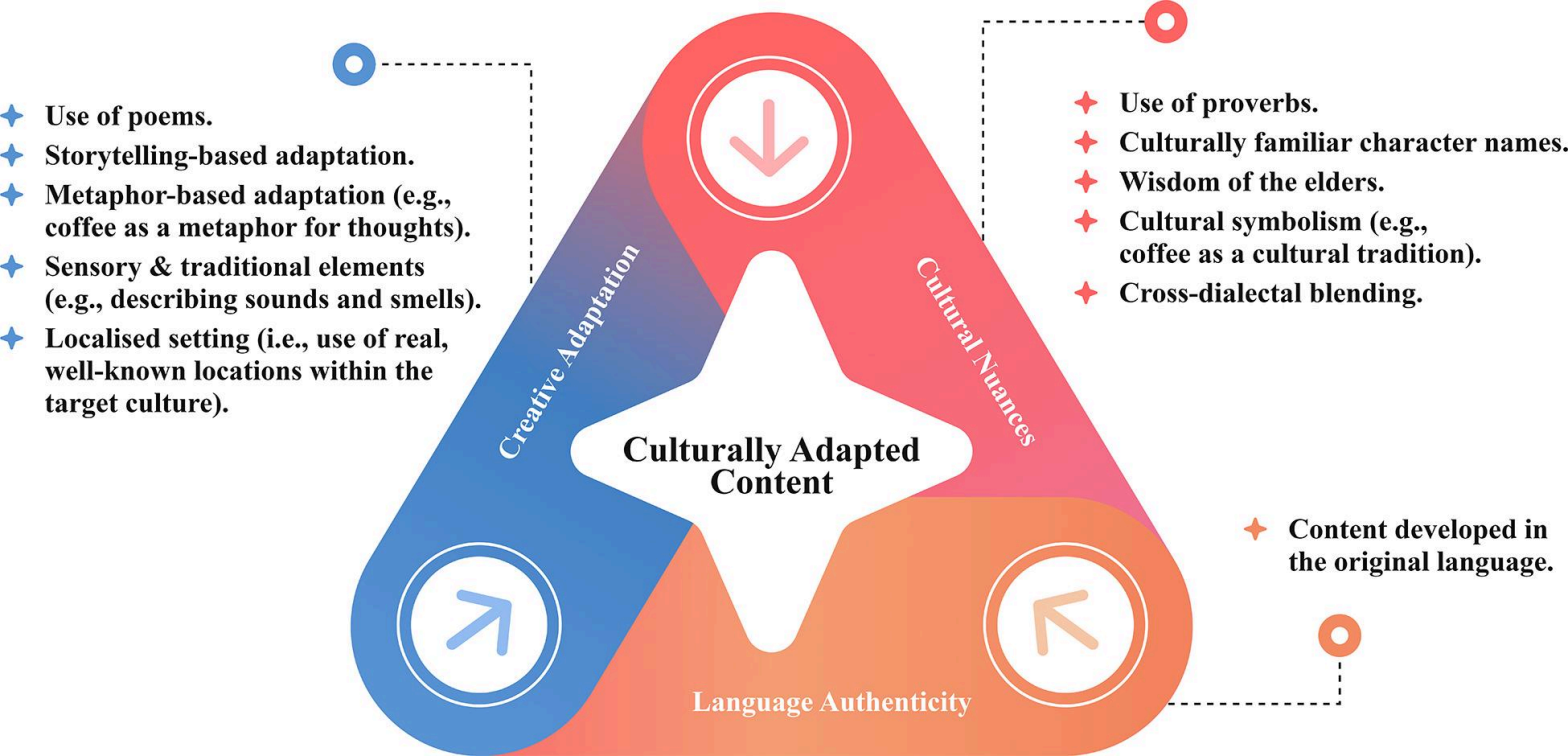
Strategies and techniques for cultural adoption of content.

The cultural content adoption strategy employs three main dimensions (see Figure 4): *Language Authenticity*, *Cultural Nuances*, and *Creative Adaptation*. To be culturally appropriate, content should be written in the original language, reviewed by native speakers, and then translated into English to reflect the intended audience's language, ensuring natural flow, idiomatic precision, and authentic expression. The interventions then go beyond mechanical translation and use culturally centred language developed in the target community's native language (66). Cultural characteristics can be emphasised through techniques such as proverbs, cultural symbolism (such as coffee in Arabic society), and culturally familiar names, all of which build an instant connection with readers (57, 67). For those with religious-based cultures or strong spiritual traditions, expressions of such beliefs, such as verses, can be integrated to support the content's message, ensuring their spiritual significance is maintained and contextualised (67, 69). Another technique that can be employed is the use of elder wisdom, especially relevant to collectivist cultures. Using an elder mentor figure to model time values and to demonstrate respect for wisdom and learning through example or guidance reinforces cultural authority and the relational bonds that are central to collectivist societies (82).

Furthermore, cross-dialectal blending can be employed by including commonly recognised expressions and terms to make the content more accessible to subcultures. The language used in interventions can be customised not just to a cultural group as a whole, but also to regional and subcultural dialects, emphasising authenticity (66). These factors represent the cultural context and ground the content in the target culture's language, dialects, traditions, heritage, and values (*Cultural Nuances*). Additionally, artistic components such as poems, storytelling-based adaptations, and metaphor-based adaptations, sensory and traditional elements (such as the aroma of coffee) can be incorporated as an innovative means to immerse the reader in a familiar cultural setting and to introduce complex psychological concepts through an engaging narrative (57, 88, 89), making abstract ideas easier to grasp (*Creative Adaptation*). These approaches can combine to provide a richly tailored, culturally immersive experience, making the content engaging, accessible, and relevant, which helps overcome barriers such as stigma, distrust, or cultural distance that might otherwise limit user engagement.

### 3.4 UX/UI adaptation

The fourth layer emphasises the importance of culturally responsive UI/UX design, which combines the structure and flow of user experience (UX) with the visual and symbolic elements of the interface. UX adaptation entails tailoring elements such as navigation, flow timing and pacing, content structure, and feedback to meet cultural expectations for interaction and communication (75). For instance, in collectivistic cultures, users may respond better to structured, linear pathways with clearly defined steps and progress indicators, reflecting cultural preferences for guided experiences and hierarchical organisation (73, 90). In contrast, users from more individualistic cultures may prefer flexible navigation when exploring content (73, 90). Similarly, in collectivist or faith-based contexts, longer engagement periods are more acceptable. Designing for reflection, storytelling, or gradual task progression, rather than efficiency-driven interactions that prioritise speed and task switching, can foster a greater sense of connection (73, 91). Visual and interactive elements, such as colours, icons, layouts, and text orientation, must all be culturally appropriate and familiar (73–75). These UX and UI adaptations ensure the digital experience is usable, meaningful, and engaging.

**Figure 5:**
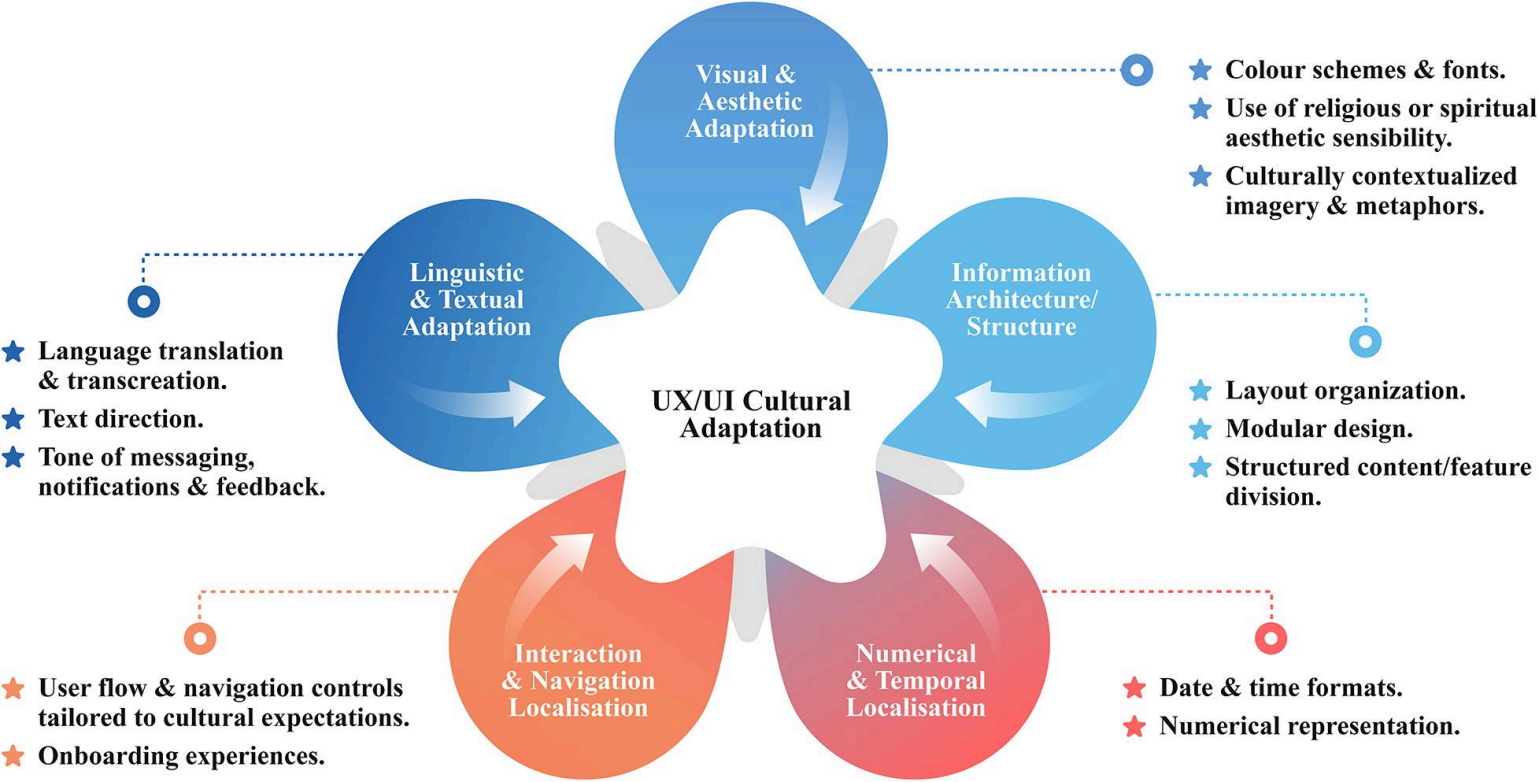
Cultural adaptation approaches for UX/UI design.

UX/UI can be culturally adapted, for example, by using cultural values and norms and drawing on established research frameworks (73, 74) as guidelines to inform the process. As shown in Figure 5, cultural adaptation involves a diverse approach to ensure the design corresponds with the target users. *Linguistic & Textual Adaptation* includes language translation, transcreation, and, if applicable, the appropriate text direction. Error messages and notifications are tailored with culturally relevant language and tones to promote clarity*. Visual & Aesthetic Adaptation* selects colour schemes and fonts following local cultural preferences. Religious design elements and culturally contextualised imagery and metaphors are incorporated to boost local relevance. For *Interaction & Navigation Localisation*, a well-structured, modular layout with discrete sections provides predictability and clear navigation pathways, especially for collectivist societies.

Additionally, welcome screens may use familiar language and expressions to provide a culturally appropriate onboarding experience. *Information architecture and structure, especially important for collectivist societies,* can be used to organise content and features into readily apparent components and clearly defined sections, making it easier for users to understand and interact with various elements. Lastly, the *Numerical & Temporal Localisation* dimension can utilise culturally relevant dates and number formats, including a timer, utilising culture-specific numerals.

## 4 Co-design as a strategy for culturally adaptive mental health interventions

Co-design has emerged as a major method for cultural adaptation, enabling members of minority groups with unique cultural contexts to shape interventions based on their lived experiences. While co-design is a powerful approach to involve end-users, especially those with lived experience, it is challenging because it requires iterative feedback and the integration of diverse perspectives. Keeping participants engaged throughout the co-design can be difficult, and stigma associated with mental health issues and concerns about confidentiality are the main barriers to mental health help-seeking and accessing mental health care (92).

Additionally, standardising co-design approaches is unlikely within culturally and linguistically diverse (CALD) communities, as protocols developed within a cultural framework may not account for the unique needs, values, and contexts of others. Emphasised, therefore, is the need to adopt culturally responsive procedures that recognise and understand cultural differences within the target group (93, 94). The experiences of CALD communities during co-design are influenced by the quality of relationships with researchers (95). Strong relationships promote open communication and trust, which are essential for effective collaboration. Long-term collaborations ensure that the changing needs and perspectives of CALD communities are considered (95).

Involving end users during design promotes theoretically sound, practical, and beneficial mental health care. A low uptake of technology-based interventions could be caused by a failure to use design processes that consider end-user perspectives (96–98). In co-design, tailoring interventions, increasing engagement, identifying needs, and empowering participants are the primary benefits of involving individuals with lived experience (99). Standard top-down interventions might overlook cultural nuances (100). Instead, cultural nuances are integrated using a bottom-up co-design approach, placing the target community at the heart of the design process to bridge gaps and address challenges that would otherwise be overlooked (101). Co-designing mental mHealth interventions may help support people with depression among collectivist and religious-based CALD communities. This is because values, beliefs, language, mental health literacy, stigma, social factors, and cultural nuances all affect the acceptance and efficacy of mental health interventions (102, 103).

In co-design, users' lived experiences and cultural contexts contribute to the relevance and effectiveness of interventions, thereby minimising the need for extensive post-development adaptations. Conducting an e-survey before co-design workshops may help recruit participants and capture initial perspectives, providing context that shapes focus group discussions (101, 102). Key themes, user needs, and design considerations can be identified and explored in depth during the workshops. Paired with existing research frameworks (12, 67, 69–71) and evidence from systematic review of existing culturally focussed mental health mHealth apps (104), which, as Robinson et al. (105) emphasise, are crucial for identifying existing evidence and its gaps, these early stage findings can be used to create an initial targeted prototype. This preparatory work is designed to encourage valuable participation in co-design workshops by focusing on unmet needs, avoiding duplication, and ensuring efficient, effective sessions through targeted activities. This sequential yet overlapping methodology is designed to produce a comprehensive collection of quantitative and qualitative data, enabling a well-informed, user-centred design.

### 4.1 Laying the groundwork: initial prototype for co-design

**Figure 6:**
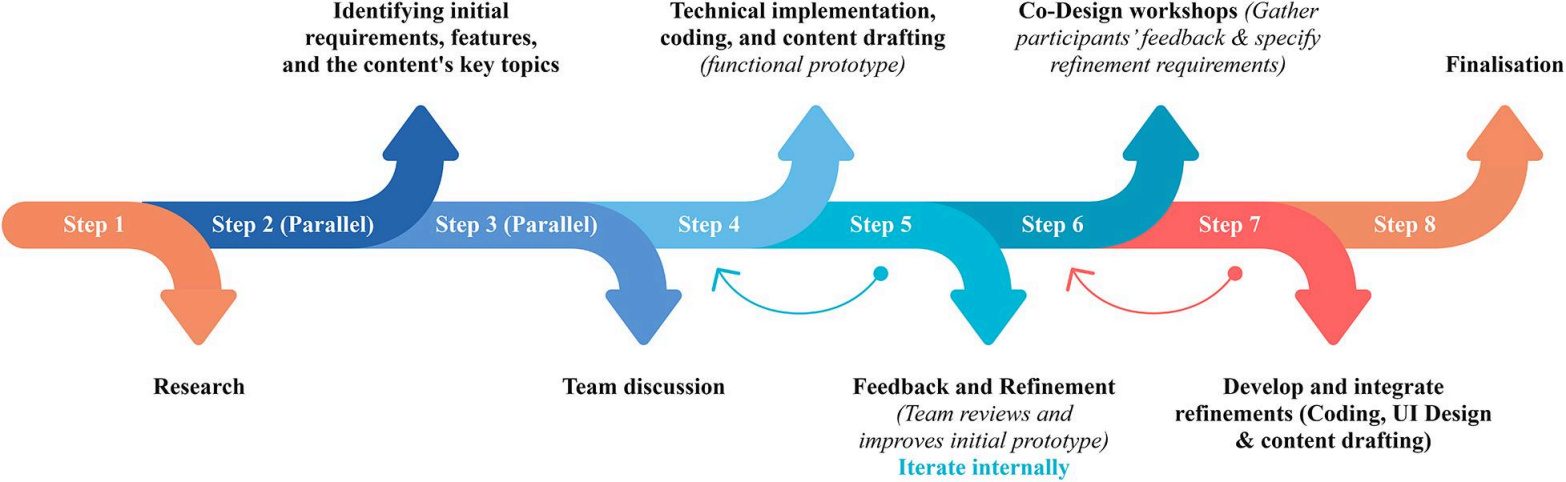
Process for codesigning prototype.

Experience prototyping, as defined by Buchenau and Fulton Suri (106), embodies an approach to crafting integrated experiences and serves three vital functions: understanding existing user experiences and contexts, exploring and evaluating design ideas, and communicating concepts to stakeholders. By presenting a prototype at the outset, facilitators can provide a foundation for discussion and feedback, identify tacit needs, and accelerate creative ideation and iterative development. In workshops, users can interact directly with the prototype to provide insights. Feedback from prototypes can be used for iterative improvement, ensuring the app aligns with the target user's needs. Figure 6 shows a proposed stepwise approach to prototype design.

Integrating lived experience perspectives in the prototype goes beyond the UI design and the features or tools offered. Participants can play a large role in developing content, particularly psychoeducational modules and resources, as well as concept explanations, ensuring their perspectives are embedded in the co-design. Such involvement leads to better outputs, relevance and varied perspectives. Ege et al. (107) noted that “the most successful strategy focused on learning about the problem or solution space, often via physical prototypes rather than following more prescriptive ‘theoretical' methodologies”, which could emphasise the importance of ongoing internal prototyping. Given this, we recommend developing and evaluating a quick internal prototype to define the intervention's components. The steps then expose and challenge the development team's preconceptions before engaging end users. This prototype can provide a tangible starting point that guides but does not constrain the co-design process, ensuring sessions remain focused and adaptable. Consequently, when end users are engaged, they collaborate on thoughtful, iterated concepts rather than abstract ideas, resulting in deeper involvement and culturally aligned outputs.

As shown in Figure 6, key topics based on project goals and reviews of existing frameworks should be identified to co-design content or resources. A team meeting is ideal for discussing and finalising these topics, ensuring they align with the project's objectives and the target audience's needs. After identifying the key topics, scripts for the initial content are written and can be reviewed by external psychology and CBT experts, who can discuss the content with other authors to ensure it is grounded in evidence and informed by expert knowledge and best practices. The prototype's content is then developed to incorporate cultural adaptation techniques to optimise relatability and engagement. An iterative process includes feedback and suggestions to refine and align the prototype's concept, features, design, and content with a shared vision. The prototype then serves as a starting point for co-design workshops, where participants' personal feedback and creative input are used to refine and expand the prototype iteratively. Participants can propose new ideas for interactive features, tools, content, and formats. This collaborative approach ensures the prototype is culturally relevant and highly effective in achieving the intended outcomes.

### 4.2 Developing personas to encourage better engagement

Stigma can create barriers to co-design participation for vulnerable populations (108), as it may lead to feelings of shame or fear of judgment, underscoring the need for tailored approaches or innovative methods to address this specific barrier. Persona is a user modelling technique that communicates an audience's needs and behaviours, providing insight into their preferences and behaviours (109). As an effective co-design technique for culturally and linguistically diverse groups (110), personas can reduce the effects of stigma by shifting the focus away from individuals themselves and making participants feel that discussions are not personally directed at them. Co-created personas can help users with diverse needs participate more meaningfully in co-design by making the design process more inclusive, accessible, and reflective of broader user experiences (111).

The initial personas can be designed using information gleaned from the literature review and e-survey to encapsulate key traits and represent familiar attitudes and circumstances of the target community; personas are then validated, enhanced, and extended during co-design workshops. Personas can serve as conversation starters during workshops to reduce feelings of isolation and stimulate open dialogue, as participants are encouraged to express their thoughts and emotions more freely while discussing the persona's actions or responses.

## 5 Limitations

While the proposed framework offers valuable guidance for developing culturally responsive digital mental health interventions, particularly regarding sequencing and the depth of adaptation, it has certain limitations. Notably, it does not address challenges unique to digital contexts, such as privacy and data security, which might be crucial for user engagement. In many cultural contexts, concerns about confidentiality and stigma may deter individuals from using digital platforms (112, 113), especially when protective measures are unclear or incompatible with local expectations (108). To resolve these matters, Zhang et al. (114) suggested that mobile mental health systems must tailor their safeguards to the sensitivity of each data type, prioritising comprehensive protections for sharing and transmission over mere collection or storage. To address individuals' varying privacy concerns, solutions should be user-centred and customisable. This includes offering granular controls (e.g., selective sharing, data retraction, adjustable privacy tiers), as well as measures such as data minimisation, deidentification, end-to-end encryption, and continuous authentication. Equally important is building in routine audits and risk assessments, inquiring about any past privacy breaches to recommend tailored safeguards, and educating users about how their information is managed and secured (114). Furthermore, while co-design is a powerful strategy for achieving deep cultural adaptation for specific populations, a potential peril is that this approach may limit the generalisability of the resulting design to broader populations. Building a platform that is truly responsive to a wide array of diverse populations would necessitate significant resources for repeated co-design processes and iterative adaptations, posing a challenge for broader scalability.

Two further limitations, moreover, are that the framework does not address broader structural barriers, such as unequal access to digital technologies and low levels of technology literacy, which can substantially affect the reach of digital tools (112, 115). Technologically, unequal mHealth access and connectivity challenges can be reduced by using an offline-first, low-bandwidth architecture (113), in which all fundamental functions, such as mood recording, guided exercises, and psychoeducation, are stored locally and synchronised only when a reliable connection is available. This ensures that users with intermittent connectivity or limited data plans can continue to use apps. On a social level, community-based “digital navigators” can bridge the digital literacy gap (113), providing hands-on training, real-time support, and modelling of essential workflows. These strategies can support mHealth technologies to remain both technically resilient and practically accessible. These limitations underscore the pressing need for future research to integrate cultural adaptation, digital inclusion, and ethical implementation, and to translate these insights into next-generation mHealth frameworks that foster fair, accessible, and long-term engagement across diverse contexts.

## 6 Discussion and conclusion

Despite the rapid expansion of digital mental health interventions, many existing mHealth solutions continue to inadequately address cultural context, often treating adaptation as a superficial or post-development step. Designing culturally adapted digital interventions entails more than simply translating or adjusting visual elements; these adaptations must be embedded in the underlying logic, interaction flow, and overall layout of the user experience in the digital environment.

We have proposed a framework to effectively guide the development and scaling of culturally responsive digital iCBT interventions, integrating the adaptation of core therapeutic mechanisms with culturally attuned interaction and UX/UI design. This framework marks an advance over previous models by offering a comprehensive, deeply integrated approach to cultural adaptation, specifically tailored to the digital environment. An example of this innovation is process of explicitly incorporating religious factors with practical guidance for their implementation within digital health interventions. This framework moves beyond the limitations of earlier frameworks, which often lacked digital context, focused on superficial adaptations, or did not explicitly address religious dimensions.

Cultural responsiveness is integrated directly into the entire digital development lifecycle of mHealth apps. This model posits a novel, integrated four-layer approach: 1) cultural adaptation of the therapeutic foundation; 2) culturally grounding features and reframing standard tools; 3) content and messaging adaptation; and 4) UX (User Experience)/UI (User Interface) adaptation. All layers are anchored by a participatory, community-led co-design and prototyping methodology, ensuring cultural insights are directly incorporated from lived experiences rather than being imposed top-down.

A co-design strategy that engages members of the target community in developing the intervention from the outset is vital for achieving cultural adaptation of digital interventions across all the described layers. Instead of implementing cultural adaptations from the outside, co-design ensures cultural insights are grounded in participants' experiences, values, and preferences. An initial prototype enables co-design, providing a solid starting point for reflection, feedback, and creativity. The prototype gives participants something tangible to respond to and reshape rather than requiring them to develop ideas from scratch, which can be intimidating or overwhelming, especially for those without design or technical expertise. This technique boosts confidence, promotes deeper participation, and facilitates more focused and innovative inputs. It also helps researchers, designers, and participants communicate more effectively by establishing a shared visual language.

In conclusion, the proposed framework supports cultural adaptation throughout the intervention design through co-design and prototyping, resulting in meaningful, culturally grounded digital solutions. While developed for iCBT interventions, this framework can be easily adapted to other digital health applications and serve as a guide for tailoring interventions to diverse populations.

## Data Availability

The original contributions presented in the study are included in the article/Supplementary Material, further inquiries can be directed to the corresponding author.

## References

[1] Linardon J Cuijpers P Carlbring P Messer M Fuller-Tyszkiewicz M. The efficacy of app-supported smartphone interventions for mental health problems: a meta-analysis of randomized controlled trials. World Psychiatry. (2019) 18(3):325–36. 10.1002/wps.20673 31496095 PMC6732686

[2] Firth J Torous J Nicholas J Carney R Pratap A Rosenbaum S The efficacy of smartphone-based mental health interventions for depressive symptoms: a meta-analysis of randomized controlled trials. World Psychiatry. (2017) 16(3):287–98. 10.1002/wps.20472 28941113 PMC5608852

[3] Chandrashekar P. Do mental health mobile apps work: evidence and recommendations for designing high-efficacy mental health mobile apps. Mhealth. (2018) 4:6. 10.21037/mhealth.2018.03.02 29682510 PMC5897664

[4] Price M Yuen EK Goetter EM Herbert JD Forman EM Acierno R Mhealth: a mechanism to deliver more accessible, more effective mental health care. Clin Psychol Psychother. (2014) 21(5):427–36. 10.1002/cpp.1855 23918764 PMC3926903

[5] Olff M. Mobile mental health: a challenging research agenda. Eur J Psychotraumatol. (2015) 6(1):27882. 10.3402/ejpt.v6.27882 25994025 PMC4439418

[6] Ellis DM Draheim AA Anderson PL. Culturally adapted digital mental health interventions for ethnic/racial minorities: a systematic review and meta-analysis. J Consult Clin Psychol. (2022) 90(10):717. 10.1037/ccp0000759 36227330

[7] Schueller SM Hunter JF Figueroa C Aguilera A. Use of digital mental health for marginalized and underservedpopulations. Curr Treat Options Psychiatry. (2019) 6:243–55. 10.1007/s40501-019-00181-z

[8] Eyles H Jull A Dobson R Firestone R Whittaker R Te Morenga L Co-design of mHealth delivered interventions: a systematic review to assess key methods and processes. Curr Nutr Rep. (2016) 5:160–7. 10.1007/s13668-016-0165-7

[9] Willis HA Gonzalez JC Call CC Quezada D. Scholars for elevating equity and diversity (SEED), galán CA.culturally responsive telepsychology & mHealth interventions for racial-ethnic minoritized youth: research gaps and future directions. J Clin Child Adolesc Psychol. (2022) 51(6):1053–69. 10.1080/15374416.2022.2124516 36227174 PMC9627988

[10] Jackson DN Sehgal N Baur C. Benefits of mHealth co-design for African American and hispanic adults: multi-method participatory research for a health information app. JMIR Formative Research. (2022) 6(3):e26764. 10.2196/26764 35262496 PMC8943540

[11] Shahsavar Y Choudhury A. Effectiveness of evidence based mental health apps on user health outcome: a systematic literature review. PLoS One. (2025) 20(3):e0319983. 10.1371/journal.pone.0319983 40131942 PMC11936281

[12] Naeem F Phiri P Rathod S Ayub M. Cultural adaptation of cognitive–behavioural therapy. BJPsych Adv. (2019) 25(6):387–95. 10.1192/bja.2019.15

[13] Teater B. Cognitive behavioural therapy (CBT). In: Davies M, editor. The Blackwell Companion to Social Work. Hoboken, NJ: Wiley Blackwell (2013). p. 423–27.

[14] Gratzer D Khalid-Khan F. Internet-delivered cognitive behavioural therapy in the treatment of psychiatric illness. Cmaj. (2016) 188(4):263–72. 10.1503/cmaj.150007 26527829 PMC4771536

[15] Lindegaard T Brohede D Koshnaw K Osman SS Johansson R Andersson G. Internet-based treatment of depressive symptoms in a kurdish population: a randomized controlled trial. J Clin Psychol. (2019) 75(6):985–98. 10.1002/jclp.22753 30702758

[16] Eylem O van Straten A de Wit L Rathod S Bhui K Kerkhof AJFM. Reducing suicidal ideation among turkish migrants in The Netherlands and in the UK: the feasibility of a randomised controlled trial of a guided online intervention. Pilot Feasibility Stud. (2021) 7:1–18. 10.1186/s40814-021-00772-9 33494831 PMC7830826

[17] De Jesús-Romero R Holder-Dixon AR Buss JF Lorenzo-Luaces L. Race, ethnicity, and other cultural background factors in trials of internet-based cognitive behavioral therapy for depression: systematic review. J Med Internet Res. (2024) 26:e50780. 10.2196/50780 38300699 PMC10870215

[18] Abudabbeh N Hays PA. Cognitive-behavioral therapy with people of Arab heritage. In: Hays PA Iwamasa GY, editors. *Culturally Responsive Cognitive-Behavioral Therapy: Assessment, Practice, and Supervision.*. Washington, DC: American Psychological Association (2006). p. 141–59. 10.1037/11433-006

[19] Kayrouz R Dear BF Kayrouz B Karin E Gandy M Titov N. Meta-analysis of the efficacy and acceptability of cognitivebehavioural therapy for arab adult populations experiencing anxiety, depression or post-traumatic stress disorder. Cogn Behav Ther. (2018) 47(5):412–30. 10.1080/16506073.2018.1445124 29714106

[20] Thomas J Ashraf S. Exploring the Islamic tradition for resonance and dissonance with cognitive therapy for depression. Ment Health Relig Cult. (2011) 14(2):183–90. 10.1080/13674676.2010.517190

[21] Abdelati NS. The effectiveness of Islamic cognitive behavioral therapy with selected Islamic content for depressed adults in Libya (Unpublished Doctorate dissertation). Penang: Universiti Sains Malaysia (2016).

[22] Beshai S Clark CM Dobson KS. Conceptual and pragmatic considerations in the use of cognitive-behavioral therapy with Muslim clients. Cognit Ther Res. (2013) 37:197–206. 10.1007/s10608-012-9450-y

[23] Rathod S Phiri P Naeem F. An evidence-based framework to culturally adapt cognitive behaviour therapy. The Cognitive Behaviour Therapist. (2019) 12:e10. 10.1017/S1754470X18000247

[24] Eskici HS Hinton DE Jalal B Yurtbakan T Acarturk C. Culturally adapted cognitive behavioral therapy for Syrian refugee women in Turkey: a randomized controlled trial. Psychol Trauma. (2023) 15(2):189. 10.1037/tra0001138 34618479

[25] Carlson KM González-Prendes AA. Cognitive behavioral therapy with religious and spiritual clients: a critical perspective. J Spiritual Ment Health. (2016) 18(4):253–82. 10.1080/19349637.2016.1159940

[26] Paukert AL Phillips L Cully JA Loboprabhu SM Lomax JW Stanley MA. Integration of religion into cognitive-behavioral therapy for geriatric anxiety and depression. J Psychiatr Pract. (2009) 15(2):103–12. 10.1097/01.pra.0000348363.88676.4d 19339844

[27] Chowdhary N Jotheeswaran AT Nadkarni A Hollon SD King M Jordans MJD The methods and outcomes of cultural adaptations of psychological treatments for depressive disorders: a systematic review. Psychol Med. (2014) 44(6):1131–46. 10.1017/S0033291713001785 23866176 PMC3943384

[28] Bernal G Jiménez-Chafey MI Domenech Rodríguez MM. Cultural adaptation of treatments: a resource for considering culture in evidence-based practice. Prof Psychol Res Pract. (2009) 40(4):361. 10.1037/a0016401

[29] Rochelle NS Hoyer J. A cross-cultural conceptual comparison of behavioral activation and Ikigai. J Contemp Psychother. (2024) 54(1):37–46. 10.1007/s10879-023-09592-9

[30] Husain N Lunat F Lovell K Miah J Chew-Graham CA Bee P Efficacy of a culturally adapted, cognitive behavioural therapy based intervention for postnatal depression in British south Asian women (ROSHNI-2): a multicentre, randomised controlled trial. Lancet. (2024) 404(10461):1430–43. 10.1016/S0140-6736(24)01612-X 39396350

[31] Husain A Hodge DR. Islamically modified cognitive behavioral therapy: enhancing outcomes by increasing the cultural congruence of cognitive behavioral therapy self-statements. Int Soc Work. (2016) 59(3):393–405. 10.1177/0020872816629193

[32] Naeem F Sarhandi I Gul M Khalid M Aslam M Anbrin A A multicentre randomised controlled trial of a carer supervised culturally adapted CBT (CaCBT) based self-help for depression in Pakistan. J Affect Disord. (2014) 156:224–27. 10.1016/j.jad.2013.10.051 24274963

[33] Greimel KV Kröner-Herwig B. Cognitive behavioral treatment (CBT). In: Møller AR Langguth B De Ridder D Kleinjung T, editors. Textbook of Tinnitus. New York, NY: Springer (2011). p. 557–61. 10.1007/978-1-60761-145-5_71

[34] Pineros-Leano M Cintrón V Piedra LM. Culturally adapted cognitive interventions for depression: treatment tools from Vida Alegre. In: Benuto L. editor. Toolkit for Counseling Spanish-Speaking Clients. Cham: Springer. (2017). p. 221–43. 10.1007/978-3-319-64880-4_10

[35] Faber J Lee E. Cognitive-behavioral therapy for a refugee mother with depression and anxiety. Clin Case Stud. (2020) 19(4):239–57. 10.1177/1534650120924128

[36] Hwang W-C Wood JJ Lin K-M Cheung F. Cognitive-behavioral therapy with Chinese Americans: research, theory, and clinical practice. Cogn Behav Pract. (2006) 13(4):293–303. 10.1016/j.cbpra.2006.04.010

[37] Chartier IS Provencher MD. Behavioural activation for depression: efficacy, effectiveness and dissemination. J Affect Disord. (2013) 145(3):292–99. 10.1016/j.jad.2012.07.023 22884236

[38] Ekers D Webster L Van Straten A Cuijpers P Richards D Gilbody S. Behavioural activation for depression; an update of meta-analysis of effectiveness and sub group analysis. PLoS One. (2014) 9(6):e100100. 10.1371/journal.pone.0100100 24936656 PMC4061095

[39] Soucy I Provencher M Fortier M McFadden T. Efficacy of guided self-help behavioural activation and physical activity for depression: a randomized controlled trial. Cogn Behav Ther. (2017) 46(6):493–506. 10.1080/16506073.2017.1337806 28644740

[40] Stein AT Carl E Cuijpers P Karyotaki E Smits JAJ. Looking beyond depression: a meta-analysis of the effect ofbehavioral activation on depression, anxiety, and activation. Psychol Med. (2021) 51(9):1491–504. 10.1017/S0033291720000239 32138802

[41] Lehmann DC Boerdlein C. A systematic review of culturally adapted behavioral activation treatments for depression. Research on Social Work Practice. (2020) 30(6):688–702. 10.1177/1049731520915635

[42] Bait Jameel SA. The effectiveness of low intensity cognitive behavioral therapy (LI-CBT) on reducing symptoms of depression in Arab clients. (2017).

[43] Hopko DR Lejuez CW Ruggiero KJ Eifert GH. Contemporary behavioral activation treatments for depression: procedures, principles, and progress. Clin Psychol Rev. (2003) 23(5):699–717. 10.1016/S0272-7358(03)00070-9 12971906

[44] Benson-Flórez G Santiago-Rivera A Nagy G. Culturally adapted behavioral activation: a treatment approach for a latino family. Clin Case Stud. (2017) 16(1):9–24. 10.1177/1534650116668630

[45] Mir G West R Meer S Rabbee J Song N. Evaluating a Culturally Adapted Behavioural Activation Therapy (BA-M). Leeds: University of Leeds (2023).

[46] Emmons RA Stern R. Gratitude as a psychotherapeutic intervention. J Clin Psychol. (2013) 69(8):846–55. 10.1002/jclp.22020 23775470

[47] Rash JA Matsuba MK Prkachin KM. Gratitude and well-being: who benefits the most from a gratitude intervention? Appl Psychol Health Well Being. (2011) 3(3):350–69. 10.1111/j.1758-0854.2011.01058.x

[48] Sansone RA Sansone LA. Gratitude and well being: the benefits of appreciation. Psychiatry (Edgmont). (2010) 7(11):18. 21191529 PMC3010965

[49] Chalmiers MA Istemi F Simsek S. Gratitude to god and its psychological benefits in Islamic contexts: a systematic review of the literature. Ment Health Relig Cult. (2023) 26(5):405–17. 10.1080/13674676.2022.2046714

[50] Mason O Hargreaves I. A qualitative study of mindfulness-based cognitive therapy for depression. Br J Med Psychol. (2001) 74(2):197–212. 10.1348/000711201160911 11802836

[51] MacKenzie MB Kocovski NL. Mindfulness-based cognitive therapy for depression: trends and developments. Psychol Res Behav Manag. (2016) 9:125–32. 10.2147/PRBM.S63949 27274325 PMC4876939

[52] Abdulkerim N Li C. How applicable are mindfulness-based interventions to Muslim clients in the US? Prof Psychol Res Pract. (2022) 53(3):253.

[53] Blignault I Saab H Woodland L Mannan H Kaur A. Effectiveness of a community-based group mindfulness program tailored for arabic and bangla-speaking migrants. Int J Ment Health Syst. (2021) 15(1):1–13. 10.1186/s13033-021-00456-0 33849610 PMC8042358

[54] Menon S Katona C Glover N. The effectiveness and acceptability of culturally adapted cognitive behavioural therapy for traumatised refugees and asylum seekers: a systematic review. Ment Health Sci. (2024) 2(4):e85. 10.1002/mhs2.85

[55] Praptomojati A Icanervilia AV Nauta MH Bouman TK. A systematic review of culturally adapted cognitive behavioral therapy (CA-CBT) for anxiety disorders in Southeast Asia. Asian J Psychiatr. (2024) 92:103896. 10.1016/j.ajp.2023.103896 38199202

[56] Spanhel K Balci S Feldhahn F Bengel J Baumeister H Sander LB. Cultural adaptation of internet-and mobile-based interventions for mental disorders: a systematic review. NPJ Digit Med. (2021) 4(1):128. 10.1038/s41746-021-00498-1 34433875 PMC8387403

[57] Naderbagi A Loblay V Zahed I Ekambareshwar M Poulsen A Song Y Cultural and contextual adaptation of digital health interventions: narrative review. J Med Internet Res. (2024) 26:e55130. 10.2196/55130 38980719 PMC11267096

[58] Burbach FR. Family based interventions in psychosis—an overview of, and comparison between, family therapy and family management approaches. J Ment Health. (1996) 5(2):149–64. 10.1080/09638239650037018

[59] Falloon IR. Family interventions for mental disorders: efficacy and effectiveness. World Psychiatry. (2003) 2(1):20. 16946881 PMC1525058

[60] Pharoah F Mari JJ Rathbone J Wong W. Family intervention for schizophrenia. Cochrane Database Syst Rev. (2010) 12:CD000088. 10.1002/14651858.CD000088.pub2 17054127

[61] Pilling S Bebbington P Kuipers E Garety P Geddes J Orbach G. Psychological treatments in schizophrenia: I. Meta-analysis of family intervention and cognitive behaviour therapy. Psychol Med. (2002) 32(5):763–82. 10.1017/S0033291702005895 12171372

[62] Luciano M Del Vecchio V Giacco D De Rosa C Malangone C Fiorillo A. A “family affair”? the impact of family psychoeducational interventions on depression. Expert Rev Neurother. (2012) 12(1):83–92. 10.1586/ern.11.131 22243046

[63] Heim E Kohrt BA. Cultural adaptation of scalable psychological interventions. Clin Psychol Eur. (2019) 1(4):1–22. 10.32872/cpe.v1i4.37679

[64] Bernal G Bonilla J Bellido C. Ecological validity and cultural sensitivity for outcome research: issues for the cultural adaptation and development of psychosocial treatments with Hispanics. J Abnorm Child Psychol. (1995) 23:67–82. 10.1007/BF01447045 7759675

[65] Resnicow K Soler R Braithwaite RL Ahluwalia JS Butler J. Cultural sensitivity in substance use prevention. J Community Psychol. (2000) 28(3):271–90. 10.1002/(SICI)1520-6629(200005)28:3<271::AID-JCOP4>3.0.CO;2-I

[66] Bernal G Sáez-Santiago E. Culturally centered psychosocial interventions. J Community Psychol. (2006) 34(2):121–32. 10.1002/jcop.20096 PMC11571183 39563877

[67] Hinton DE Patel A. Cultural adaptations of cognitive behavioral therapy. Psychiatr Clin. (2017) 40(4):701–14. 10.1016/j.psc.2017.08.006 29080595

[68] Barrera M Jr Castro FG. A heuristic framework for the cultural adaptation of interventions. Clin Psychol Sci Pract. (2006) 13(4):311–6. 10.1111/j.1468-2850.2006.00043.x

[69] Jalal B Samir SW Hinton DE. Adaptation of CBT for traumatized Egyptians: examples from culturally adapted CBT (CA-CBT). Cogn Behav Pract. (2017) 24(1):58–71. 10.1016/j.cbpra.2016.03.001

[70] Algahtani HMS Almulhim A AlNajjar FA Ali MK Irfan M Ayub M Cultural adaptation of cognitive behavioural therapy (CBT) for patients with depression and anxiety in Saudi Arabia and Bahrain: a qualitative study exploring views of patients, carers, and mental health professionals. Cogn Behav Ther. (2019) 12:e44. 10.1017/S1754470X1900028X

[71] Almoshmosh N Jefee Bahloul H Barkil-Oteo A Hassan G Kirmayer LJ. Mental health of resettled Syrian refugees: a practical cross-cultural guide for practitioners. J Ment Health Train Educ Pract. (2020) 15(1):20–32. 10.1108/JMHTEP-03-2019-0013

[72] Martinengo L Stona AC Griva K Dazzan P Pariante CM von Wangenheim F Self-guided cognitive behavioral therapy apps for depression: systematic assessment of features, functionality, and congruence with evidence. J Med Internet Res. (2021) 23(7):e27619. 10.2196/27619 34328431 PMC8367167

[73] Marcus A Gould EW. Crosscurrents: cultural dimensions and global web user-interface design. Interactions. (2000) 7(4):32–46. 10.1145/345190.345238

[74] Alsswey AH Al-Samarraie H El-Qirem FA Alzahrani AI Alfarraj O. Culture in the design of mHealth UI: an effort to increase acceptance among culturally specific groups. Electron Libr. (2020) 38(2):257–72. 10.1108/EL-04-2019-0097

[75] Vergari F. Navigating cultural diversity in UI/UX design of mobile applications: A design approach. (2024).

[76] Kunorubwe T. Cultural adaptations of group CBT for depressed clients from diverse backgrounds: a systematic review. The Cognitive Behaviour Therapist. (2023) 16:e35. 10.1017/S1754470X23000302

[77] Lim C Sim K Renjan V Sam HF Quah SL. Adapted cognitive-behavioral therapy for religious individuals with mental disorder: a systematic review. Asian J Psychiatr. (2014) 9:3–12. 10.1016/j.ajp.2013.12.011 24813028

[78] de Abreu Costa M Moreira-Almeida A. Religion-adapted cognitive behavioral therapy: a review and description of techniques. J Relig Health. (2022) 61(1):443–66. 10.1007/s10943-021-01345-z 34518980 PMC8837510

[79] Pearce MJ Koenig HG Robins CJ Nelson B Shaw SF Cohen HJ Religiously integrated cognitive behavioral therapy: a new method of treatment for major depression in patients with chronic medical illness. Psychotherapy. (2015) 52(1):56. 10.1037/a0036448 25365155 PMC4457450

[80] Erwahyudin DD. Adapting technology in Islamic psychology: exploring digital pathways to spiritual and psychological wellbeing. In: Pambuko ZB Setiyo M Praja CBE Setiawan A Yuliastuti F Muliawanti L, et al., editors. Proceedings of 5th Borobudur International Symposium on Humanities and Social Science 2023, Advances in Social Science, Education and Humanities Research, Vol. 856. Dordrecht: Atlantis Press (2024). p. 745–54. 10.2991/978-2-38476-273-6_78

[81] Zannat N Mahmud M. The development of an Islamic-inspired digital therapeutics app: exploring mental health challenges among university students. Int J Softw Eng Comput Syst. (2025) 11(1):70–91. 10.15282/ijsecs.11.1.2025.6.0138

[82] Dardas LA Al-leimon O Gladstone T Dabbas AA Alammouri I Van Voorhees B. Validating a digital depression prevention program for adolescents in Jordan: cultural adaptation and user testing in a randomized controlled trial. Front Psychiatry. (2025) 16:1529006. 10.3389/fpsyt.2025.1529006 40012714 PMC11860973

[83] Burchert S Alkneme MS Bird M Carswell K Cuijpers P Hansen P User-centered app adaptation of a low-intensity e-mental health intervention for Syrian refugees. Front Psychiatry. (2019) 9:663. 10.3389/fpsyt.2018.00663 30740065 PMC6355704

[84] Demetry Y Wasteson E Lindegaard T Abuleil A Geranmayeh A Andersson G Individually tailored and culturally adapted internet-based cognitive behavioral therapy for arabic-speaking youths with mental health problems in Sweden: qualitative feasibility study. JMIR Form Res. (2023) 7(1):e46253. 10.2196/46253 37999955 PMC10709795

[85] Alnaghaimshi NIS Awadalla MS Proeve M Clark SR Baumert M. Cultural adaptation mental mhealth apps–qualitative analysis of co-design workshops involving Arabic-speaking communities. (2025). OSF Preprints. 10.31234/osf.io/fmcbw_v1

[86] Khan S Lovell K Lunat F Masood Y Shah S Tomenson B Culturally-adapted cognitive behavioural therapy based intervention for maternal depression: a mixed-methods feasibility study. BMC Womens Health. (2019) 19:1–11. 10.1186/s12905-019-0712-7 30691431 PMC6350293

[87] Noar SM Benac CN Harris MS. Does tailoring matter? Meta-analytic review of tailored print health behavior change interventions. Psychol Bull. (2007) 133(4):673. 10.1037/0033-2909.133.4.673 17592961

[88] McCall B Shallcross L Wilson M Fuller C Hayward A. Storytelling as a research tool used to explore insights and as an intervention in public health: a systematic narrative review. Int J Public Health. (2021) 66:1604262. 10.3389/ijph.2021.1604262 34795554 PMC8592844

[89] Asner-Self KK Feyissa A. The use of poetry in psychoeducational groups with multicultural-multilingual clients. J Spec Group Work. (2002) 27(2):136–60. 10.1177/0193392202027002003

[90] Broeder P Gkogka A. The cultural impact of navigation design in global e-commerce. J Tour Herit Serv Mark. (2020) 6(3):46–53.

[91] Kim H Coyle JR Gould SJ. Collectivist and individualist influences on website design in South Korea and the US: a cross-cultural content analysis. J Comput Mediat Commun. (2009) 14(3):581–601. 10.1111/j.1083-6101.2009.01454.x

[92] Kiselev N Pfaltz M Haas F Schick M Kappen M Sijbrandij M Structural and socio-cultural barriers to accessing mental healthcare among Syrian refugees and asylum seekers in Switzerland. Eur J Psychotraumatol. (2020) 11(1):1717825. 10.1080/20008198.2020.1717825 32128044 PMC7034440

[93] Langdon SE Golden SL Arnold EM Maynor RF Bryant A Freeman VK Lessons learned from a community-based participatory research mental health promotion program for American Indian youth. Health Promot Pract. (2016) 17(3):457–63. 10.1177/1524839916636568 27009131 PMC9097107

[94] Novins DK Boyd ML Brotherton DT Fickenscher A Moore L Spicer P. Walking on: celebrating the journeys of native American adolescents with substance use problems on the winding road to healing. J Psychoact Drugs. (2012) 44(2):153–59. 10.1080/02791072.2012.684628 22880543

[95] O’Brien J Fossey E Palmer VJ. A scoping review of the use of co-design methods with culturally and linguistically diverse communities to improve or adapt mental health services. Health Soc Care Community. (2021) 29(1):1–17. 10.1111/hsc.13105 32686881

[96] Torous J Nicholas J Larsen ME Firth J Christensen H. Clinical review of user engagement with mental health smartphone apps: evidence, theory and improvements. BMJ Ment Health. (2018) 21(3):116–19. 10.1136/eb-2018-102891 PMC10270395 29871870

[97] Birnbaum F Lewis DM Rosen R Ranney ML. Patient engagement and the design of digital health. Acad Emerg Med. (2015) 22(6):754. 10.1111/acem.12692 25997375 PMC4674428

[98] Vial S Boudhraâ S Dumont M. Human-centered design approaches in digital mental health interventions: exploratory mapping review. JMIR Ment Health. (2022) 9(6):e35591. 10.2196/35591 35671081 PMC9214621

[99] Veldmeijer L Terlouw G Van Os J Van Dijk O Van ‘t Veer J Boonstra N. The involvement of service users and people with lived experience in mental health care innovation through design: systematic review. JMIR Ment Health. (2023) 10(1):e46590. 10.2196/46590 37490326 PMC10410372

[100] Hall GCN Berkman ET Zane NW Leong FTL Hwang W-C Nezu AM Reducing mental health disparities by increasing the personal relevance of interventions. Am Psychol. (2021) 76(1):91. 10.1037/amp0000616 32118456 PMC8034200

[101] Sanders EBN Stappers PJ. Co-creation and the new landscapes of design. CoDesign. (2008) 4(1):5–18. 10.1080/15710880701875068

[102] Turner AE Cheng HL Llamas JD Tran GTTA Hill XK Fretts MJ Factors impacting the current trends in the use of outpatient psychiatric treatment among diverse ethnic groups. Curr Psychiatry Rev. (2016) 12(2):199–220. 10.2174/1573400512666160216234524

[103] Saechao F Sharrock S Reicherter D Livingston JD Aylward A Whisnant J Stressors and barriers to using mental health services among diverse groups of first-generation immigrants to the United States. Community Ment Health J. (2012) 48:98–106. 10.1007/s10597-011-9419-4 21655942

[104] Alnaghaimshi NIS Awadalla MS Clark SR Baumert M. A systematic review of features and content quality of arabic mental mHealth apps. Front Digit Health. (2024) 6:1472251. 10.3389/fdgth.2024.1472251 39723151 PMC11668747

[105] Robinson KA Saldanha IJ Mckoy NA. Development of a framework to identify research gaps from systematic reviews. J Clin Epidemiol. (2011) 64(12):1325–30. 10.1016/j.jclinepi.2011.06.009 21937195

[106] Buchenau M Suri JF. Experience prototyping. In: Proceedings of the 3rd Conference on Designing Interactive Systems: Processes, Practices, Methods, and Techniques. New York, NY: Association for Computing Machinery (2000). p. 424–33. 10.1145/347642.347802

[107] Ege DN Goudswaard M Gopsill J Steinert M Hicks B. What, how and when should I prototype? An empirical study of design team prototyping practices at the IDEA challenge hackathon. Des Sci. (2024) 10:e22. 10.1017/dsj.2024.16

[108] Aljedaani B Ahmad A Zahedi M Babar MA. Security awareness of end-users of mobile health applications: an empirical study. In: MobiQuitous 2020 – 17th EAI International Conference on Mobile and Ubiquitous Systems: Computing, Networking and Services. New York, NY: Association for Computing Machinery (2021). p. 125–36. 10.1145/3448891.3448952

[109] Alsaadi B Alahmadi D. The use of persona towards human-centered design in health field: review of types and technologies. In: 2021 International Conference on e-Health and Bioengineering (EHB). Piscataway, NJ: IEEE (2021). p. 1–4. 10.1109/EHB52898.2021.9657744

[110] Ali PA Salway S Such E Dearden A Willox M. Enhancing health literacy through co-design: development of culturally appropriate materials on genetic risk and customary consanguineous marriage. Prim Health Care Res Dev. (2019) 20:e2. 10.1017/S1463423618000038 29642973 PMC6476369

[111] Neate T Bourazeri A Roper A Stumpf S Wilson S. Co-created personas: engaging and empowering users with diverse needs within the design process. In: Proceedings of the 2019 CHI Conference on Human Factors in Computing Systems. New York, NY: Association for Computing Machinery (2019). p. 1–12. 10.1145/3290605.3300880

[112] Estrela M Semedo G Roque F Ferreira PL Herdeiro MT. Sociodemographic determinants of digital health literacy: a systematic review and meta-analysis. Int J Med Inf. (2023) 177:105124. 10.1016/j.ijmedinf.2023.105124 37329766

[113] Wisniewski H Torous J. Digital navigators to implement smartphone and digital tools in care. Acta Psychiatr Scand. (2020) 141(4):350–55. 10.1111/acps.13149 31930477 PMC7928068

[114] Zhang D Lim J Zhou L Dahl AA. Breaking the data value-privacy paradox in mobile mental health systems through user-centered privacy protection: a web-based survey study. JMIR Ment Health. (2021) 8(12):e31633. 10.2196/31633 34951604 PMC8742208

[115] Arias López MDP Ong BA Borrat Frigola X Fernández AL Hicklent RS Obeles AJT Digital literacy as a new determinant of health: a scoping review. PLOS Digit Health. (2023) 2(10):e0000279. 10.1371/journal.pdig.0000279 37824584 PMC10569540

